# No increased prevalence of autoantibodies neutralizing type I IFNs in idiopathic pulmonary fibrosis patients

**DOI:** 10.1186/s12931-023-02396-4

**Published:** 2023-03-20

**Authors:** Quentin Philippot, Paul Bastard, Anne Puel, Jean-Laurent Casanova, Aurélie Cobat, Cédric Laouénan, Coralie Tardivon, Bruno Crestani, Raphael Borie

**Affiliations:** 1 Laboratory of Human Genetics of Infectious Diseases, Institut National de la Santé et de la Recherche Médicale (INSERM) U1163, Necker Hospital for Sick Children, Necker Branch, Paris, France; 2 Imagine Institute, University of Paris, Paris, France; 3 St. Giles Laboratory of Human Genetics of Infectious Diseases, Rockefeller Branch, Rockefeller University, New York, NY USA; 4 Pediatric Hematology-Immunology and Rheumatology Unit, Necker Hospital for Sick Children, Assistante Publique-Hôpitaux de Paris (AP-HP), Paris, France; 5 Department of Pediatrics, Necker Hospital for Sick Children, Paris, France; 6 Howard Hughes Medical Institute, New York, NY USA; 7 Inserm, IAME, Université Paris Cité and Université Sorbonne Paris Nord, Paris, France; 8 Département d’Epidémiologie Biostatistiques et Recherche Clinique, AP-HP, Hôpital Bichat, Paris, France; 9 Service de Pneumologie A Hôpital Bichat, APHP, 46 Rue Henri Huchard, 75877 Paris CEDEX 18, France; 10 Inserm, PHERE, Université Paris Cité, 75018 Paris, France

**Keywords:** COVID-19, Idiopathic pulmonary fibrosis, Autoimmunity

## Abstract

SARS-CoV2 infection has a poor prognosis in patients affected of idiopathic pulmonary fibrosis (IPF). Autoantibodies (auto-Abs) neutralizing type I interferons (IFNs) are found in the blood of at least 15% of patients with life-threatening COVID-19 pneumonia. Because of the elevated prevalence of some auto-Abs in IPF patients, we hypothesize that the prevalence of auto-Abs neutralizing type I IFNs might be increased in the IPF population and then explained specific poor outcome after COVID-19. We screened the plasma of 247 consecutive IPF patients for the presence of auto-Abs neutralizing type I IFNs. Three patients displayed auto-Abs neutralizing type I IFNs. Among them, the only patient with documented SARS-CoV-2 infection experienced life threatening COVID-19 pneumonia. The prevalence of auto-Abs neutralizing type I IFNs in this cohort of IPF patients was not significantly different from the one of the general population. Overall, this study did not suggest any association between auto-Abs neutralizing type I IFNs and IPF.

## To the editor,

Respiratory virus infections in patients affected of idiopathic pulmonary fibrosis (IPF) may trigger acute exacerbation [1–4]. Among them, SARS-CoV2 infection has a poor prognosis, with reported mortality rate of up to 57% [2, 3, 5]. Autoantibodies (auto-Abs) neutralizing type I interferons (IFN) have been found in at least 15% of patients with life threatening COVID-19 pneumonias [6]. These auto-Abs are associated with life-threatening COVID-19 pneumonia, with odd-ratios (OR) increasing with the number and concentration of type I IFNs neutralized (with ORs ranging from 3 to 67) [6, 7]. Though the diagnosis of IPF requires the exclusion of a connective tissue disease, auto-Abs, such as antineutrophil cytoplasmic or antiperiplakin auto-Abs may be present in up to 40% of IPF patients [8]. We hypothesize that the prevalence of auto-Abs neutralizing type I IFNs might be increased in the IPF populations and then explained specific poor outcome after COVID-19. We therefore aimed to (1) assess the prevalence of auto-Abs neutralizing type I IFNs in IPF patients compared to the general population and (2) analyze the medical history of IPF patients with auto-Abs neutralizing type I IFNs.

247 IPF patients were prospectively and consecutively recruited in our center between November 2010 and June 2019. At IPF diagnosis, given in agreement with the ATS/ERS/JRS/ALAT guidelines [9], a plasma sample was obtained for each patient. Auto-Abs against IFN-α2 and ω were assessed by Gyros. The neutralizing activity of auto-Abs against IFN-α, ω and β was studied by a luciferase assay as previously reported [6]. The prevalence of auto-Abs neutralizing IFN-α2, ω and β in IPF patients was then compared to their prevalence in 36,775 individuals from the general population reported by Bastard et al*.* by means of Firth’s bias-corrected logistic regression as implemented in the “logistf” R package (https://rdrr.io/cran/logistf/) and adjusting for age and sex [6, 10]. This study was approved by the “Comité de Protection des Personnes Ile de France 1” (n° 0911932) and was conducted in accordance with the Helsinki Declaration. Written informed consent was obtained for all the participants.

At inclusion, the mean age was 71 ± 9 years, with a majority of men (79%), the mean forced vital capacity (FVC) was 80 ± 23% and diffusing capacity for carbon monoxide (DLCO) was 47 ± 17% of predicted value. During follow-up, 141 (57%) patients received antifibrotic drugs: pirfenidone in 62 patients (25%), nintedanib in 47 patients (19%), and both drugs in 32 patients (13%). The mean survival was 63 months. In univariate analysis (Cox model), increased age (Hazard ratio (HR) = 1.03 [95% confidence interval (CI): 1.01–10.05], p = 0.002), male sex (HR = 1.54 [1.01–2.36], p = 0.04), lower FVC (HR = 0.98 [0.97–0.99], p < 0.001) and DLCO (HR = 0.95 [0.94–0.97], p < 0.001) at inclusion were significantly associated with a reduced survival. Only three patients had auto-Abs neutralizing type I IFNs and the global prevalence of auto-Abs neutralizing type I IFNs in IPF patients did not significantly differ from their prevalence in the general population adjusted for the age and the sex (Table 1).

**Table 1:** Comparison of the prevalence of auto-Abs to specific sets of type I IFNs in IPF patients to that of 36,775 individuals from the general population, adjusted on age and sex

Auto-Abs neutralizing type I IFN positive (amount of type I IFNs neutralized, in plasma diluted 1:10)	OR [95% CI]	p
Auto-Abs neutralizing IFNα2 and ω (10 ng/ml)	0.57 [0.00–4.01]	0.66
Auto-Abs neutralizing IFNα2 or ω (10 ng/ml)	0.50 [0.06–1.81]	0.34
Auto-Abs neutralizing IFNα2 (10 ng/ml)	0.71 [0.08–2.61]	0.66
Auto-Abs neutralizing IFNω (10 ng/ml)	0.28 [0.00–1.94]	0.26
Auto-Abs neutralizing IFN-β (10 ng/ml)	2.32 [0.25–9.68]	0.38
Auto-Abs neutralizing IFNα2 and ω (100 pg/ml)	0.71 [0.08–2.63]	0.66
Auto-Abs neutralizing IFNα2 or ω (100 pg/ml)	0.54 [0.15–1.35]	0.21
Auto-Abs neutralizing IFNα2 (100 pg/ml)	0.55 [0.15–1.35]	0.31
Auto-Abs neutralizing IFNω (100 pg/ml)	0.61 [0.13–1.76]	0.40

Odds ratio (OR) and p values were estimated by means of Firth’s bias-corrected logistic regression

The first patient was a 62-year-old male at IPF diagnosis of mild severity. He was treated successively with pirfenidone and nintedanib. He was still alive 5 years after inclusion and did not present symptomatic COVID-19, acute exacerbation or severe infection during his follow-up. The second patient was a 63-year-old male at the diagnosis of severe IPF. He was treated with pirfenidone. He died after 11 months of respiratory failure from end-stage pulmonary fibrosis without any documented infection. The third patient was a 76-year-old male at IPF diagnosis of mild severity, in December 2014. He was treated with nintedanib. He presented a severe COVID-19 infection in December 2020 (50% of compromised lung), received high flow nasal cannula therapy and dexamethasone for 10 days allowing him to be released from the intensive care and then from the hospital. However, he presented a *Mycobacterium bovis* pulmonary infection and a significant radiological progression of the lung fibrosis, a worsening of the dyspnea and the pulmonary function test. He died in March 2022 of end-stage lung fibrosis.

This study does not suggest any increased prevalence of auto-Abs neutralizing type I IFNs in IPF patients. The presence of auto-Abs neutralizing type I IFNs, in the three patients was not associated with any particular characteristics at diagnosis. Only one of these three patients had a diagnosis of SARS-CoV-2 infection almost 6 years after the initial IPF diagnosis. In line with the increased risk of life threatening COVID-19 pneumonia in patient with Auto-Abs to type I IFNs, and in patients with IPF [2, 3, 5], he developed a life threatening COVID-19 pneumonia and ultimately died 3 months after the infection. The monocentric and retrospective design of our study is a limitation. However, our cohort is representative of a real-life cohort from a tertiary care center without unexpected prevalence of young patients referred for lung transplantation or familial pulmonary fibrosis for instance. The low ethnic diversity in our cohort, with few patients of Asiatic ancestry, is another limitation of this study. As the prevalence of auto-Abs increased with age, some patients from this cohort have probably acquired auto-Abs neutralizing type I IFNs during follow-up [6]. The assessment for auto-Abs neutralizing type I IFNs only at IPF diagnosis is consequently a limit of this study and the effect of antifibrotic on auto-Abs production cannot be evaluated. In conclusion, this study does not suggest any association between auto-Abs neutralizing type I IFNs and IPF.

## Data Availability

All raw and processed data are available upon request from the corresponding authors under a data transfer agreement.

## Abbreviations

Auto-Abs: Autoantibodies
IFN: Interferon
IPF: Idiopathic pulmonary fibrosis
OR: Odd Ratio
FVC: Forced vital capacity
DLCO: Diffusing capacity for carbon monoxide
